# Supplementary material for: SyNDI: synchronous network data integration framework
Source: BMC Bioinformatics. 2018 Nov 6;19:403. doi: 10.1186/s12859-018-2426-5 (PMC6219086; doi:10.1186/s12859-018-2426-5)
Supplement: Supplementary file 5 — Files of the example presented in Additional file 4. This additional file is a zip package that contains all files used in Additional file 4. Detailed descriptions of each of these files are presented in a README file included in this zip package. (GZ 30411 kb) [file 12859_2018_2426_MOESM5_ESM.gz › SyNDITools/Meme2Fimo/meme_4.9.0_4.tar.gz!/meme_4.9.0/website/html/help_multiple_genomes.html]

MEME Suite - Multiple Genomes


|  |  |
| --- | --- |
|  | MEME Suite The MEME Suite web application requires the use of JavaScript   Javascript doesn't seem to be available on your browser.  Multiple genomes  Some MEME Suite applications can utilize data from multiple genomes to increase the sensitivity of the analysis via comparative genomics. In this case, sequences in the primary genome, along with orthologous sequences from a set of comparative genomes, are used by the application in its final analysis.  When the multiple genomes option is available, a fixed set of comparative genomes is used by the MEME Suite application. You can view the identities of these genomes by clicking on "supported database" on the application's input web form. The types of sequences (e.g., length, location with respect to TSS etc.) are the same as for the primary genome. |
